# Transfer learning approach based on computed tomography images for predicting late xerostomia after radiotherapy in patients with oropharyngeal cancer

**DOI:** 10.3389/fmed.2022.993395

**Published:** 2022-09-23

**Authors:** Annarita Fanizzi, Giovanni Scognamillo, Alessandra Nestola, Santa Bambace, Samantha Bove, Maria Colomba Comes, Cristian Cristofaro, Vittorio Didonna, Alessia Di Rito, Angelo Errico, Loredana Palermo, Pasquale Tamborra, Michele Troiano, Salvatore Parisi, Rossella Villani, Alfredo Zito, Marco Lioce, Raffaella Massafra

**Affiliations:** ^1^IRCCS Istituto Tumori “Giovanni Paolo II,” Bari, Italy; ^2^Ospedale Monsignor Raffaele Dimiccoli, Barletta, Italy; ^3^IRCCS Casa Sollievo della Sofferenza, Opera di San Pio da Pietrelcina Viale Cappuccini, Foggia, Italy

**Keywords:** deep learning, xerostomia, oropharyngeal cancer, CT images, CNN–convolutional neural network

## Abstract

**Background and purpose:**

Although the latest breakthroughs in radiotherapy (RT) techniques have led to a decrease in adverse event rates, these techniques are still associated with substantial toxicity, including xerostomia. Imaging biomarkers could be useful to predict the toxicity risk related to each individual patient. Our preliminary work aims to develop a radiomic-based support tool exploiting pre-treatment CT images to predict late xerostomia risk in 3 months after RT in patients with oropharyngeal cancer (OPC).

**Materials and methods:**

We performed a multicenter data collection. We enrolled 61 patients referred to three care centers in Apulia, Italy, out of which 22 patients experienced at least mild xerostomia 3 months after the end of the RT cycle. Pre-treatment CT images, clinical and dose features, and alcohol-smoking habits were collected. We proposed a transfer learning approach to extract quantitative imaging features from CT images by means of a pre-trained convolutional neural network (CNN) architecture. An optimal feature subset was then identified to train an SVM classifier. To evaluate the robustness of the proposed model with respect to different manual contouring practices on CTs, we repeated the same image analysis pipeline on “fake” parotid contours.

**Results:**

The best performances were achieved by the model exploiting the radiomic features alone. On the independent test, the model reached median AUC, accuracy, sensitivity, and specificity values of 81.17, 83.33, 71.43, and 90.91%, respectively. The model was robust with respect to diverse manual parotid contouring procedures.

**Conclusion:**

Radiomic analysis could help to develop a valid support tool for clinicians in planning radiotherapy treatment, by providing a risk score of the toxicity development for each individual patient, thus improving the quality of life of the same patient, without compromising patient care.

## Introduction

Oropharyngeal squamous cell carcinomas (OPCs) are tumors that could be located in the soft palate, the pharyngeal wall, the tonsils, or the base of tongue (1).

Treatment-related toxicity is a significant problem due to the close proximity of the tumor mass to normal tissues and organs. Modern radiotherapy techniques, such as volumetric modulated arc therapy (VMAT) or intensity modulation radiotherapy (IMRT), have overcome the conventional techniques, in attempting to reduce the toxicities induced by radiation (2).

Nonetheless, RT treatments are still associated with severe toxicity, including dysphagia, mucositis, and xerostomia. In particular, xerostomia, i.e., dryness of the oral cavity caused by reduced or absent saliva flow, is common late toxicity that negatively affects patients’ quality of life either by impairing speech or swallowing or even chewing (3). This toxicity occurs especially when median doses above 26 Gy are applied to both parotids with the volume irradiated above a patient-individual threshold which is probably the most relevant predictive parameter (4, 5).

An accurate and personalized prediction of radiation-induced toxicity could support clinicians in planning an optimal treatment path. Although radiation-induced xerostomia mainly results from damage to the major salivary glands that are usually included in radiation fields, other factors are notoriously associated with the likelihood of developing toxicity in the parotids, such as parotid volume, parotid eccentricity heterogeneity, salivary gland density, amount of predisposed fat, etc. Recently, several radiomic-based models have been proposed for the prediction of late xerostomia in patients with head and neck cancer, also achieving promising performances. They showed that there is a personal risk factor for developing toxicity related to the texture of the organs at risk (OARs). Typically, most of these methods are based on the designing of the so-called handcrafted features, which have a physical meaning of the measure being considered. More recently, cutting-edge deep learning models have been used to automatically extract more sophisticated and higher-level hierarchical characteristics (6–9). These features can be lost in interpretation because they are extracted from images that undergo many processing and convolution steps, but allow the evaluation of finer and informative characteristics that cannot be quantified on the original image. Models trained on radiomic features extracted from computed tomography (CT)/magnetic resonance imaging (stocktickerMRI) and combined with clinical and dose characteristics have recently been proposed for predicting toxicity in head and neck tumors (10–14).

To the best of our knowledge, the xerostomia predictive models proposed in the literature are designed for head and neck tumors which include several locations anatomical sites of the primary tumor. There is a lack of models tailored for patients with OPC (15, 16). Compared to treatment in other areas of the head and neck, the oropharynx represents the most frequently treated site for which the definition of a plan that preserves the functionality of the parotid is more complex (17, 18). Therefore, in this work, we proposed a transfer learning approach for the definition of an accurate radiomic-based model trained on pre-treatment CT with the goal of predicting late xerostomia in patients with OPC. The radiomic features were extracted by using a pre-trained convolutional neural network (CNN) and subsequently processed by different state-of-the-art machine learning algorithms (19–21).

We also evaluated the predictive power of dosimetric parameters and clinical features, both separately and in conjunction with radiomic features. Furthermore, since the contouring of both OARs and the target is an operator-dependent process, we have investigated the strength of the model with respect to the manual contouring processes of the parotid. The results obtained were achieved on a multicenter dataset and validated both in cross-validation and on an independent set.

## Materials and methods

### Enrolled patients and collected data

For this study, we performed a multicenter data collection. We enrolled 61 patients from Apulia, Italy, out of which 32 patients were referred to Istituto Tumori “Giovanni Paolo II” in Bari (Apulia, Italy), 15 patients to Casa Sollievo della Sofferenza Hospital in San Giovanni Rotondo (Apulia, Italy), and 14 patients to “Monsignor Raffaele Dimiccoli” Hospital in Barletta (Apulia, Italy). Patients were enrolled according to the following criteria:

• histologic diagnosis of squamous cell carcinoma of the oropharynx
• treatment with primary radiotherapy, with or without concomitant chemotherapy or cetuximab,
• follow-up period (with the evaluation of xerostomia) of at least 3 months,
• availability of pre-treatment CT.

All patients were consecutively included in a data registration program as part of routine clinical practice. The study was approved by the Institutional Review Board of Istituto Tumori “Giovanni Paolo II” Bari, Italy (Approval Code: 24269/21). All the centers involved in the study signed a data transfer agreement.

The collected clinical features were: age at diagnosis, tumor size (T: T1a, T1b, T1c, T2, T3, T4), lymph nodes stage (N: 0, 1, 2, 3), surgery (Yes/NO), induction chemotherapy (induction CHT: Yes, No), concurrent CHT during RT (concurrent CHT: Yes, No), platinum-based CHT (Yes/NO), weight pre-RT (Kg), smoking history (Yes, No, Ex), and alcohol history (Yes, No, Ex). Hereinafter, this dataset consisting of 11 characteristics is referred to as the *Clinical Feature Set* (abbr. *Clin_FS*).

**FIGURE 1:**
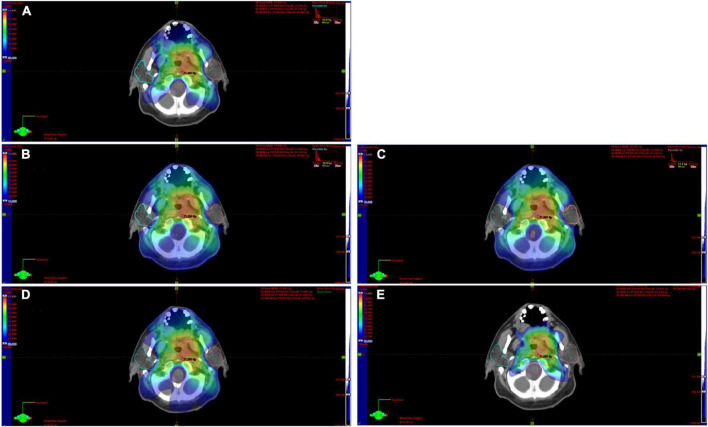
Contouring of the parotids on CT images and the related dose map. In this explanatory case, both the left and right parotid showed a D20 equal to 26.8 Gy **(A)**. The D40 of the right parotid was equal to 14.88 **(B)** the left one was 15.62 **(C)**. Panels **(D,E)** show the volume covered by an isodose of 20 and 40 Gy, respectively.

Among the enrolled 61 patients, 34 patients were treated with the VMAT RT technique, while 27 patients were treated with IMRT RT technique. All treatment plans included a simultaneous integrated boost and tried to spare a dose to the parotid glands without compromising the dose to the target volumes. For both parotids, the mean dose (left and right mean dose), volume receiving 20 and 40 Gy of radiation (left and right V20, left and right V40), and dose received by 20 and 40% of the volume (left and right D20, left and right D40) were extracted from dose-volume histograms (DVHs). Figure 1 shows the contouring of the parotids and how the dose map was overlaid to illustrate the calculation of the dose features set. Previous studies have shown that these dose features were the most important parameters in the prediction of long xerostomia after RT (22). Hereinafter, this dataset consisting of 10 dose features is referred to as the DVH Feature Set (abbr. DVH_FS).

Moreover, for each patient, a planning pre-treatment CT was acquired and used to extract radiomics features, as described in the following section.

### Radiomic feature extraction

All pre-treatment CT images were acquired at the time of simulation, prior to the beginning of the treatment. Pre-treatment CT was used for contouring and RT planning. All CT images were acquired using dedicated and customized immobilization and reproducibility systems (SIRs) (versaboard and 9-point thermoplastic mask). The pre-treatment CT series is generated by an area subtended between the keel bifurcation and the vertex of the head, using an acquisition spiral with a thickness of 3 mm with pitch equal to 1 (contiguous scans), 120 kV, and 350 mAs. The FOV used is the maximum one (600 mm) with a standard brain acquisition filter and a 512 × 512 matrix.

The parotids are contoured by expert radiotherapists of the involved Institutes. The parotids were then automatically segmented by extracting a binary mask for the structures of interest. For each patient, radiomic features were extracted by a transfer learning approach from both left and right parotids. Transfer learning approach is usually used when relatively small-size datasets are analyzed. Specifically, we made use of the high-performing pre-trained CNN, called AlexNet, as a feature extractor. AlexNet is a CNN with eight deep layers (23, 24). It has previously been trained on more than a million images to solve image classification tasks. Such a network constructs a hierarchical representation of input images: deeper layers contain higher-level features, constructed using the lower-level features of earlier layers.

The knowledge learned by the network during the training phase was here transferred to our images to extract features useful to train a classification model for predicting late xerostomia. Since AlexNet requires an image input size of 227-by-227, parotids segmentation has previously been resized to patches of this size to be given as input to the network. The radiomic features were extracted from planning DICOM files.

In this work, we extracted features from the “*pool1*” layer of the network architecture which corresponds to the first pooling layer. The “*pool1*” layer had an output with dimensions of 27 × 27 × 96 that was flattened to a single 69984-length features vector. The “*pool1*” layer is one of the initial layers of the network. Thus, the corresponding extracted features are low-level features, namely, representations of local details of an image, such as edges, dots, and curves. We extracted the features not directly from a convolution layer that returns the feature maps but after the application of pooling that, as well-known in deep learning theory, makes features invariant to truncation, occlusion, and translation (25).

The CT image of each patient is made up of a different number of 2D slides. From each slide, radiomic features were extracted by transfer learning approach, i.e., using a pre-trained network. As a result, several vectors of radiomic features, as many as the number of slices that make up the CT, are associated with each patient. To obtain only one vector radiomic feature in correspondence to each single patient, we computed the maximum value of each feature. Hence, the final vector was composed of the maximum values for each feature.

Although multicenter studies are necessary to demonstrate the potential clinical value of radiomics as a prognostic tool, the variability factors introduced by scanner models, acquisition protocols, and reconstruction settings need particular attention. Indeed, it is well-known that radiomic characteristics are very sensitive to these factors. We then applied a statistical harmonization method called ComBat which was first developed to treat the “batch effect” in gene expression microarray data and is also effectively used in radiomics-based studies (26–28).

During the analysis and evaluation of the collected data, a discrepancy was found in the contouring of the volumes of interest (targets and OARs) and the related geometric expansions of the radiotherapy planning target volume (PTV) which may depend on the extent of the disease, on partial discretion within the expansion limits defined by the guidelines and the type of pre-treatment checks adopted by the various centers (29–32).

In order to evaluate the robustness of the proposed model with respect to different manual contouring practices, we repeated the image analysis pipeline on “fake” parotid contours. To obtain these “fake” parotid contours, we changed the contour of the segmented parotids from each of the three centers, called center 1, center 2, and center 3, by applying dilation or erosion processes by 10% of the volume of interest compared to the original one.

All the analyses were performed by using MATLAB R2022a (MathWorks, Inc., Natick, MA, USA) software.

### Classification model design

**FIGURE 2:**
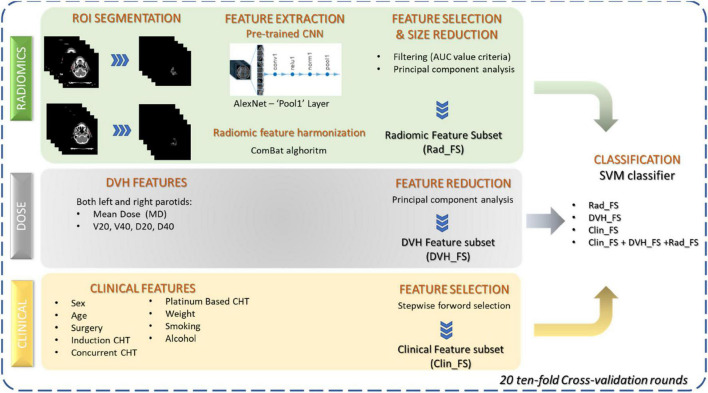
Workflow of the proposed classification approach. Three sets of features were considered: radiomic features extracted from parotid images by means of a pre-trained CNN, dose features extracted from DVH, and clinical features before RT beginning. Feature reduction and selection techniques were applied to the three sets of features to identify three subsets of significant features. SVM classifier was trained both on the individual feature subsets and using all the feature kinds jointly.

The primary objective of the present work was the prediction of xerostomia 3 months after RT in patients with OPS. As schematically illustrated in Figure 2, the classification method was developed in three phases: (i) for each dataset, a feature reduction or selection was performed, (ii) different classification models were trained on each subset of features, and (iii) finally, a classifier was trained using the selected subsets jointly.

First, a subset of the clinical feature set was selected by a sequential forward feature selection algorithm: it identified a feature subset by sequentially adding one feature at a time during a fivefold cross-validation procedure until adding more features decreases the misclassification rate of the classification model used over the same training set. Specifically, we used a discriminant analysis (33). The selected features (*Clinical Feature Subset*, *Clin_FS*) were used to train the classification model. In order to further reduce the number of selected features, we implemented a nested feature reduction technique by principal component analysis (PCA) in cross-validation (34). Only the principal components with explained variance greater than 1 were chosen (*DVH Feature Subset*, *DVH_FS*) and used to train the classification model.

A subset of radiomic feature extracted from the CT images (see section “Radiomic feature extraction”) was selected according to their discriminant power which was assessed through the computation of the area under the receiver operating curve (AUC) (35). Features whose AUC value was less than 80% were dropped from the feature radiomic set. However, these features showed a strong correlation between them. Therefore, after standardizing each feature, we implemented a nested feature reduction technique by principal component analysis (PCA) and selected the principal components with explained variance greater than one (*Radiomic Feature Subset*, *Rad_FS*) and used them to train the classification model.

The feature subsets identified are used to train a well-known machine learning algorithm, i.e., support vector machine (SVM). Specifically, we used SVM with the linear basis kernel function (36). Other classifiers known to the state of the art have been implemented but have not shown a significant performance improvement. In order not to burden the discussion, these results have not been reported either.

Finally, in order to evaluate the overall performance of all identified subsets of features, we jointly used them and trained a classification model.

A double validation of the model was carried out: (i) 20 ten-fold cross-validation rounds on 43 patients, equal to about 70% of the entire sample available and (ii) independent sample consisting of 18 patients (equal to about 30% of the entire sample available) randomly drawn and stratified with respect to the number of individual centers. The classification performances related to the iterated cross-validation procedure were evaluated in percentage terms of AUC, F-score, and accuracy, sensitivity, and specificity calculated by identifying the optimal threshold using Youden’s index on the ROC curves (37). The feature reduction or selection procedure implemented for each feature set has been nested\into the iterated cross-validation procedure. In order to evaluate the robustness of the model when the training set changes, we have calculated the same performance metrics of the same independent test set on each round of the cross-validation procedure.

### Statistical analysis and performance evaluation

The association between parotid volume of two different centers was evaluated by means of the Wilcoxon–Mann–Whitney non-parametric test (38). The same non-parametric test was used to evaluate the association between continuous features and toxicity at 3 months, whereas we used Chi-square test for those features measured on an ordinal scale (39). Correlation between continuous features was measured by Pearson’s correlation coefficient (40).

Due to the relatively small size of the sample population, a result was considered statistically significant when the *p*-value was less than 0.10 (41).

## Results

Patients’ characteristics are summarized in Table 1. A total of 61 patients with a median age at diagnosis of 59 years afferent to three different care centers was collected. Among them, 22 patients (36.07%) have shown xerostomia 3 months after RT. None of the collected clinical characteristics was statistically associated with the manifestation after 3 months from the end of the RT of the considered toxicity, except for Induction CHT (*p*-value < 0.10).

**TABLE 1:** Sample dataset characteristics.

Characteristic		Distribution	*P*-value
**Xerostomia at 3 months after RT**
	Yes (abs. %)	22 (36.07)	
	No (abs. %)	39 (69.93)	
Sex			0.52
	Male (abs. %)	47 (77.05)	
	Female (abs. %)	14 (22.95)	
Age at diagnosis			0.31
	Median (1th–3th quantile)	59.00 (54.00–68.25)	
T			0.84
	T1	2 (3.28)	
	T2	21 (34.43)	
	T3	25 (40.98)	
	T4	10 (16.39)	
	NaN	3 (4.92)	
N			0.37
	N0	6 (9.84)	
	N1	13 (21.31)	
	N2	35 (57.38)	
	N3	3 (4.92)	
	NaN	4 (6.56)	
Surgery			0.31
	Yes	53 (86.89)	
	No	8 (13.11)	
	NaN	–	
Induction CHT			0.07
	Yes	26 (42.63)	
	No	35 (57.38)	
	NaN	–	
Current CHT			0.31
	Yes	55 (90.16)	
	No	6 (9.84)	
	NaN	–	
Platinum based CHT			0.36
	Yes	52 (85.25)	
	No	7 (11.48)	
	Nan	2 (3.28)	
Weight pre-RT (Kg)			0.26
	Median (1th–3th quantile)	69.50 (60.35–80.40)	
Smoking history			0.61
	Yes	25 (40.98)	
	No	13 (21.31)	
	Ex	16 (26.23)	
	NaN	8 (13.11)	
Alcohol history			0.62
	Yes	15 (24.59)	
	No	33 (54.10)	
	Ex	1 (1.64)	
	NaN	10 (16.39)	

For categorical variables, absolute (abs.) and percentage (%) counts are reported. For continuous values, the median value and interquartile range (1st–3rd quantiles) are indicated. *P*-value related to the association test between each feature with xerostomia at 3 months after RT is shown.

### Classification performance using the parotids real contours

As described in section “Materials and methods,” an SVM classifier algorithm was trained both on the three subsets of features identified individually (*Rad_FS*, *DVH_FS*, and *Clin_FS*) and jointly. The performances of the different prediction models were evaluated both in cross-validation and on an independent test stratified random sample from the entire dataset of 61 patients.

The sample used in the cross-validation procedure consisted of 43 patients, out of which 15 patients (34.88%) had experienced xerostomia after 3 months from RT.

**FIGURE 3:**
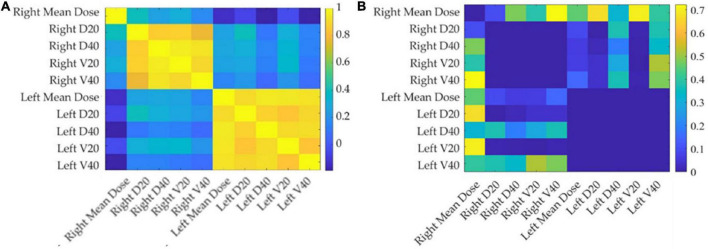
Correlation and *p*-value matrix plot of DVH features. The left panel **(A)** depicts the Pearson’s coefficients among DVH features considered in this study, while the right panel **(B)** shows the corresponding *p*-values. The DVH-extracted parotid-related dose features considered in this study show strong positive correlations.

**FIGURE 4:**
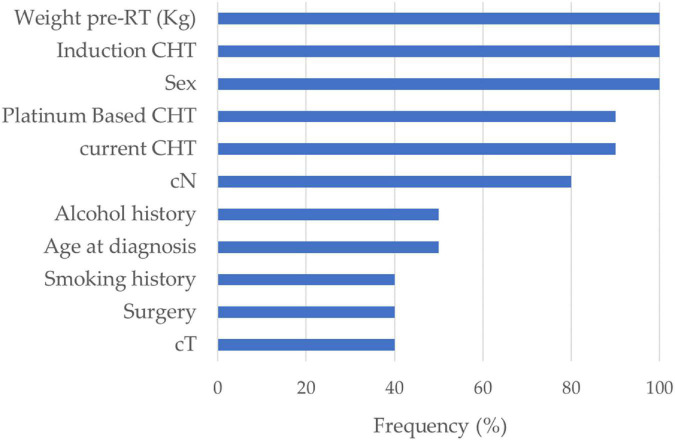
Feature selection. Statistical frequency of the clinical features selected on 20 ten-fold cross-validation rounds by means of the sequential feature selection algorithm.

Figure 3 shows the correlation among the collected DVH features: the dose features resulted as strongly correlated with each other, especially when they refer to the same area. The average number of principal components on radiomic features and selected DVH features in the different cross-validation rounds implemented were 4 and 1, respectively. Figure 4 shows the statistical frequency of the clinical features, which were selected on 20 ten-fold cross-validation procedures by means of the feature selection algorithm. The weight at the start of the RT treatment, induction CHT, and sex is the features selected with a frequency equal to 100%.

Table 2 summarizes the results achieved in cross-validation. The clinical features alone did not exceed 50%, the dose features settled around 60%, while the radiomic-based model achieved the best performances with a median value of AUC, accuracy, sensitivity, and specificity of 84.17, 88.37, 66.67, and 100%, respectively, with an F-score of 80%. The joint use of all three sets of features allows an improvement in the performance of over 5 percentage points in terms of sensitivity, reaching 73.33%.

**TABLE 2:** Classification performances of the late xerostomia predictive models in terms of median percentage and interquartile range (1st–3rd quartiles) AUC, accuracy, sensitivity, and specificity evaluated on real parotid counters.

*20 ten-fold cross-validation rounds*					

	AUC	f-score	Accuracy	Sensitivity	Specificity
*Clin_FS*	48.57 (45.00–54.76)	50.00 (48.10–50.95)	48.85 (41.86–55.81)	73.33 (53.33–80.00)	35.71 (21.43–57.14)
*DVH_FS*	59.40 (55.95–61.43)	50.33 (44.45–55.17)	69.77 (65.12–72.09)	43.33 (40.00–53.33)	80.36 (78.57–85.71)
*Rad_FS*	84.64 (84.29–86.66)	80.00 (78.57–80.00)	88.37 (86.05–88.37)	66.67 (66.67–73.33)	100 (92.86–100)
*All FS*	84.17 (82.38–85.71)	76.92 (75.86–78.57)	86.05 (83.72–86.05)	73.33 (66.67–73.33)	92.86 (89.29–96.43)
***Independent test set***					
*Clin_FS*	50.00 (42.86–56.49)	51.08 (38.75–56.00)	50.00 (38.89–61.11)	42.86 (14.29–1)	50.00 (0–72.73)
*DVH_FS*	75.97 (74.03–79.22)	62.02 (54.55–66.67)	66.67 (61.11–72.22)	42.86 (0–57.14)	90.91 (81.82–90.91)
*Rad_FS*	81.17 (79.22–81.82)	76.92 (76.92–78.69)	83.33 (83.33–83.33)	71.43 (71.43–71.43)	90.91 (90.91–90.91)
*All FS*	81.82 (81.82–88.31)	71.43 (71.43–76.92)	77.78 (77.78–77.78)	71.43 (67.14–71.43)	86.36 (81.82–90.91)

The results are evaluated both on 20 ten-fold cross-validation rounds and independent test. The related 1st and 3rd quantiles are reported in round brackets.

The proposed models were also validated on an independent sample consisting of 30% of the total sample of 61 patients. Among the 19 patients in the independent test, seven (36.84%) had experienced xerostomia 3 months after RT. The encouraging performances of the radiomic features were also confirmed on independent tests: the SVM classifier achieves an accuracy of 83.33%, a sensitivity of 71.43%, and a specificity of 90.91%. However, the improvement in sensitivity on the independent test using all three feature sets was not confirmed.

It is emphasized that both *Clin_FS* and *DVH_FS* showed a particularly variable sensitivity on the training set (53.33 and 80.00, and 40.00 and 53.33, respectively, as 1st and 3rd quantile values) and even more marked on the independent set (14.29 and 1, and 0 and 57.14, respectively, as 1 st and 3rd quantiles values).

### Classification performance using the parotid “fake” contours

**FIGURE 5:**
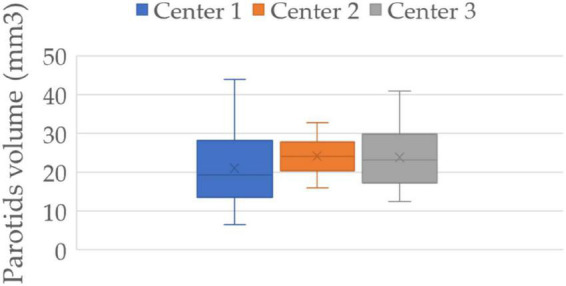
Parotids volume distribution of three centers. Center 1 shows a significantly smaller volume of the parotids than that of the other two centers (*p*-value 0.097 and 0.015), while centers 2 and 3 show no significant difference between them (*p*-value 0.575).

The contouring of the target and organs is an operator-dependent operation. The median volume and interquartile range of the three centers were 19.25 (13.65–27.8), 24.15 (20.4–27.5), and 23.19 (17.36–29.30), respectively (Figure 5). The volume distribution of center 1 differs significantly from the other two centers (*p*-value 0.097 and 0.015), while center 2 and center 3 do not show a significant difference in distribution (*p*-value 0.575). Since the most performing and stable model in external validation is the radiomic model, we wanted to evaluate the robustness of the model with respect to variations in parotid contouring. Therefore, to obtain these “fake” parotids, we dilated the volumes of patients in center 1 which showed smaller volumes on average and eroded those in centers 2 and 3 (which showed larger volumes on average) by 10% of the area of interest compared to the original one.

We then reposted the same previously proposed analysis pipeline on the parotid “fake” contours. The performances of the radiomic features still show their predictive power also following a variation of the contours of the parotids both in cross-validation and on the independent test with a median accuracy value of 81.40 and 94.44% in cross-validation and on the independent test, respectively (Table 3). It should be noted that on the independent test set, the accuracy reached using the adjusted ROI was greater than that obtained when we used the original ROI by more than 10 percentage points.

**TABLE 3:** Classification performances of the late xerostomia predictive models in terms of median percentage AUC, accuracy, sensitivity, and specificity evaluated on “fake” parotid counters.

*20 ten-fold cross-validation rounds*					

	AUC	f-score	Accuracy	Sensitivity	Specificity
*Rad_FS*	80.24 (78.93–82.14)	71.43 (71.43–74.07)	81.40 (81.40–83.72)	66.67 (66.67–66.67)	91.07 (89.29–92.86)
*All FS*	68.10 (66.90–73.10)	58.20 (55.56–60.61)	69.77 (67.44–72.09)	60.00 (53.33–66.67)	75.00 (71.43–78.57)
***Independent test set***					
*Rad_FS*	94.16 (93.51–94.81)	92.31 (83.33–92.31)	94.44 (88.89–94.44)	85.71 (71.43–85.71)	100 (100–100)
*All FS*	95.86 (88.31–970.81)	74.83 (60.00–80.00)	83.33 (77.78–83.33)	71.43 (42.86–85.71)	95.45 (81.82–100)

The results are evaluated both on 20 ten-fold cross-validation rounds and independent test. The related 1st and 3rd quantiles are reported in round brackets.

## Discussion

Radiotherapy, possibly joined with chemotherapy, represents the standard of care in patients with locally advanced oropharyngeal cancer (OPC) (42). However, RT is often associated with substantial acute and late toxicity, including xerostomia (43). Xerostomia is a frequent side effect of RT for head and neck cancer and is due to damage to the irradiated salivary glands with a relevant impact on patient s’ quality of life (44).

The latest advancement in radiotherapy techniques has improved the rate of acute adverse events in long-term survivors, yet there is a need for better identification of patients with higher risk of toxicity. In order to minimize the toxicity burden for patients with OPC, an individual toxicity risk assessment is required to adequately plan radiation treatment and any supportive therapy. Recently, computational models based on the quantitative analysis of biomedical images, i.e., radiomic analysis, have been effectively proposed to address unmet clinical needs, mainly in the field of oncological imaging (45, 46). Table 3 summarizes radiomic-based research works addressing the prediction of RT-related toxicity in head and neck patients. The models proposed at the state of the art refer in general to head and neck tumors (9–12). However, compared to treatment in other areas of the head and neck, the oropharynx represents the most frequent challenge for the preservation of radio-induced xerostomia. Therefore, the goal of our research activity was the development of a support system tailored to give an early prediction of the risk of late xerostomia after 3 months of radiotherapy treatment in patients with OPC. Specifically, we developed a deep learning-based model which exploited pre-treatment CT images. Radiomic features were extracted by a pre-trained CNN and analyzed jointly with both clinical and dose features. The usage of a transfer learning approach was here preferred to a customized CNN, i.e., to extract features and then give a prediction, because it provides some benefits especially when, as in our case, a relatively small amount of data is available. When a pre-trained network is used as a feature extractor only, no training phase is required; therefore, a drastic reduction of the computational time occurs. Moreover, for datasets counting small samples, pre-trained net allows us to obtain high generalizability of the results.

Our experimental results show that the radiomic signature has a predominant predictive potential with respect to both clinical and dose characteristics. Indeed, in the cross-validation, the radiomic features alone showed median values of AUC, accuracy, sensitivity, and specificity, 84.64, 88.37, 66.67, and 100%, respectively. The addition of the clinical and dose features only contributes to an increase in the sensitivity value (73.33%). However, this advantage on the independent test is lost, probably due to the high variability of the performances of these two data sets.

Probably, DVH_FS does not provide an added value to the prediction performance of radiomic features alone because clinicians follow the constraints defined by the guidelines in defining a treatment plan (47, 48). Rather, it seems that there is a strong predisposition to the risk of toxicity linked to the texture of the organ at risk.

The performances of the proposed radiomic model trained on CT images are encouraging if compared to the state-of-the-art models, both when trained on the same type of images (7–9) and on magnetic resonance imaging (10, 11). A classification performances overview of late xerostomia state-of-the-art predictive models is provided by Table 4. It should be emphasized that the comparison with the state of the art is purely qualitative, since in this work we considered the prediction of xerostomia at 3 months as an endpoint and the model is dedicated only to patients affected by OPC. Relevant studies currently proposed to refer to a different follow-up time and refer to the larger population of patients with head and neck cancer.

**TABLE 4:** Classification performances of the late xerostomia predictive models in terms of median percentage AUC, accuracy, sensitivity, and specificity evaluated on “*fake*” parotid counters.

References	Imaging modality	Study population and sample size	Endpoint Time of assessment	Statistics and modeling	Features	Results
MEN et al. (10)	Pre-treatment CT	784 H and N cancer patients	Xerostomia at 12th months	Model 1: 3D rCNN Model 2: Logistic Regression	3D CT 3D dose D20, V20 parotid D20, V20 submandibular Clinical data: sex, age, race, treatment arm, treatment technique, tumor site, T, N, Zubrod performance score	AUC: 0.84 Acc: 0.76 Sens: 0.76 Spec: 0.76 F-score: 0.70 AUC: 0.74 Acc: 0.64 Sens: 0.72 Spec: 0.59 F-score: 0.60
Gabryś et al. (11)	Pre-treatment CT	153 H and N cancer patients	Xerostomia at 0–6 months	Gradient tree boosting	Demographic: Age, sex 6 Handcrafted radiomics features DVH: Mean, spread, skewness	AUC: 0.65
Van Dijk et al. (12)	Pre-treatment CT	249 H and N cancer patients	Xerostomia at 12 months	Logistic regression	142 Handcrafted radiomics features DVH: Mean dose Clinical: age, sex, WHO stage, weight, length and BMI, tumor characteristics (TNM stage, tumor location) and treatment characteristics	AUC: 0.76
Sheikh et al. (13)	Pre-treatment CT MRI	249 H and N cancer patients	Xerostomia at 12 months	Generalized linear model	2877 Handcrafted radiomics features (PyRadiomics software): CT features MRI features DVH: 48 features	AUC: CLIN + CT + MR 0.73 CLIN + DVH + CT + MR 0.68
van Dijk et al. (14)	T1 weighted MRI	249 H and N cancer patients	Xerostomia at 12 months	Logistic regression	64 Handcrafted radiomics features	AUC: 0.83

The results are evaluated both on 20 ten-fold cross-validation rounds and independent test. The related 1st and 3rd quantiles are reported in round brackets.

Moreover, in this article, we also wanted to verify how robust the model was in relation to strongly operator-dependent contouring procedures. We have artificially segmented “fake” contours of the parotids and repeated the process of extracting the features and training the classification models. To the best of our knowledge, no studies for this purpose have been carried out. Even using the “fake” contours, the performances of the radiomic model are highly performing. Specifically, the results obtained using the adjusted ROI achieved very high performances in the independent test set. Our intent with the analysis of the “fake” ROI was to evaluate how much the model was still highly performing with variations on the contouring which is a notoriously operator-dependent operation.

In light of the results obtained, it would seem in fact that the erosion and dilation carried out have led to an improvement in the forecast results, that is to say, that with too many or too large contours there is a loss of information.

This result, which we have underlined in the results and discussions, offers food for thought for future works, e.g., by evaluating a forecasting model based on optimal automated segmentation.

The proposed model seems to provide reliable support regardless of the clinical contouring practice used by the operator.

Therefore, the model could accurately support clinicians in the decision-making process by providing a personal risk score for the development of toxicity, to improve the quality of life, without compromising patient care. Such a support system, if applied to clinical practice, it would allow clinicians to define a personalized radiotherapy plan by reducing the doses of the parotids as much as possible and to associate pharmacological support therapies to be carried out before and during the radiotherapy treatment.

Although our study is multicentric, the limited sample size represents a limitation of the study which, therefore, requires further validation studies. In future studies, we intend to generalize the model also for observation times and toxicities different from those considered here.

## Conclusion

In this article, we proposed a deep learning-based model to predict late toxicity after radiotherapy in patients with OPC. Specifically, we developed a radiomic-based model using pre-treatment CTs to give an early prediction of xerostomia in 3 months after RT treatment. The achieved experimental results are promising in terms of prediction accuracy. Moreover, the model is robust with respect to the manual parotid contouring procedure. Therefore, the proposed model could help to develop a valid support tool for clinicians in planning radiotherapy treatment.

## Data availability statement

The data analyzed in this study is subject to the following licenses/restrictions: The raw data supporting the conclusions of this article will be made available by the corresponding authors, without undue reservation. Requests to access these datasets should be directed to SB, s.bove@oncologico.bari.it and MC, m.c.comes@oncologico.bari.it.

## Ethics statement

The study was conducted according to the guidelines of the Declaration of Helsinki and approved by the Scientific Board of Istituto Tumori “Giovanni Paolo II,” Bari, Italy–Protocol number 24269/21. Informed consent was obtained from all subjects and/or their legal guardian(s).

## Author contributions

AF and RM: conceptualization, writing—original draft preparation, and supervision. AF, SBo, and MC: methodology. AF: software and validation. AF, GS, AN, PT, and RM: formal analysis. RM: resources. AF, GS, AN, SBa, CC, AD, AE, LP, PT, MT, SP, RV, and RM: data curation. AF, GS, AN, SBa, SBo, MC, CC, VD, AD, AE, LP, PT, MT, SP, RV, AZ, ML, and RM: writing—review and editing. All authors have read and agreed to the published version of the manuscript.
