# Corrigendum: Transfer learning approach based on computed tomography images for predicting late xerostomia after radiotherapy in patients with oropharyngeal cancer

**DOI:** 10.3389/fmed.2022.1089705

**Published:** 2022-11-22

**Authors:** Annarita Fanizzi, Giovanni Scognamillo, Alessandra Nestola, Santa Bambace, Samantha Bove, Maria Colomba Comes, Cristian Cristofaro, Vittorio Didonna, Alessia Di Rito, Angelo Errico, Loredana Palermo, Pasquale Tamborra, Michele Troiano, Salvatore Parisi, Rossella Villani, Alfredo Zito, Marco Lioce, Raffaella Massafra

**Affiliations:** ^1^IRCCS Istituto Tumori “Giovanni Paolo II”, Bari, Italy; ^2^Ospedale Monsignor Raffaele Dimiccoli, Barletta, Italy; ^3^IRCCS Casa Sollievo della Sofferenza, Opera di San Pio da Pietrelcina Viale Cappuccini, Foggia, Italy

**Keywords:** deep learning, xerostomia, oropharyngeal cancer, CT images, CNN-convolutional neural network

In the published article, there was an error in the **Funding** statement. The **Funding** statement for the Italian Ministry of Health was reported related to year “2002” as follows:

“This work was supported by funding from the Italian Ministry of Health, Ricerca Corrente 2002 Deliberation no. 219/2022, and Alleanza Contro il Cancro Association within the RADECISION project.”

The correct Funding statement appears below.

## Funding

“This work was supported by funding from the Italian Ministry of Health, Ricerca Corrente 2022 Deliberation no. 219/2022, and Alleanza Contro il Cancro Association within the RADECISION project.”

The authors apologize for this error and state that this does not change the scientific conclusions of the article in any way. The original article has been updated.

## Publisher's note

All claims expressed in this article are solely those of the authors and do not necessarily represent those of their affiliated organizations, or those of the publisher, the editors and the reviewers. Any product that may be evaluated in this article, or claim that may be made by its manufacturer, is not guaranteed or endorsed by the publisher.

